# Management of Chyloperitoneum in Laparoscopic Colorectal Surgery for Cancer: A Case Report

**DOI:** 10.1155/crgm/6690258

**Published:** 2025-08-03

**Authors:** B. Picardi, S. Rossi, F. Cortese, S. Rossi Del Monte, G. Mazzarella, S. Molica, C. Puccioni, A. D'Urso

**Affiliations:** ^1^Department of General and Emergency Surgery, San Filippo Neri Hospital, Rome, Italy; ^2^Department of Surgery, “Sapienza” University, Rome, Italy; ^3^Department of Surgery, Fondazione Policlinico A. Gemelli IRCCS, Rome, Italy

**Keywords:** chyle, chyloperitoneum, colorectal surgery, lymphadenectomy, lymphatic

## Abstract

Chyloperitoneum after colorectal surgery remains a relatively rare complication with estimated incidence of 1%–6.5%. In colorectal surgery, this complication is mostly described after D3 right colectomy. Nonoperative treatment involves several approaches. We present a rare case of chyloperitoneum after laparoscopic left hemicolectomy for left-sided colonic adenocarcinoma. A CT scan and a triglycerides dosage on the chylous liquid were performed to confirm the diagnosis. The patient was successfully treated only by nutritional measures, avoiding prolonged fasting or invasive treatment. Fasting and complete parenteral nutrition are not necessarily required in the treatment of chyle leakage.

## 1. Introduction

Chyloperitoneum (CP) after colorectal surgery remains a relatively rare complication with estimated incidence of 1%–6.5% [1]. Diagnosis is based on the typical appearance of noninfected white milky fluid from surgical drains or aspirate of (excessive) postoperative abdominal fluid or in the absence of an abdominal drain on cross-sectional postoperative imaging. It usually occurs after unrecognized iatrogenic injury to the lymphatics during surgery [1, 2]. It is reported during surgical procedures where the dissection is performed close to the lymphatic (thoracic surgeries, pancreatic resections, retroperitoneal lymph node dissection, abdominal aortic aneurysm repair, pelvic surgery in gynecology, living donor nephrectomy, and liver transplant), but nowadays, the rapid advancement in minimally invasive surgical techniques has enabled more aggressive lymphadenectomy in colorectal cancer surgery as well, in order to achieve R0 resection for better overall and disease-free survivals, for example, complete mesocolic excision (CME) is performed for right colon cancer with D3 lymphadenectomy [1, 3, 4].

Nonoperative treatment involves several approaches and is not yet standardized in the literature.

We aim to illustrate a rare case of CP after laparoscopic left hemicolectomy for left-sided colonic adenocarcinoma successfully treated only by nutritional measures, avoiding prolonged fasting or invasive treatment. The study has been written in accordance with the 2013 CARE checklist [5].

## 2. Case Presentation

A 50-year-old woman with no past medical history significant for diseases or interventions was admitted for a T1N0M0 G2 stage left-sided colonic adenocarcinoma. Laparoscopic left hemicolectomy and central vascular ligation (CVL), with an end-to-end Knight–Griffen colorectal anastomosis, was performed. Bowel perfusion was checked with IV ICG before and after completion of the anastomosis.

One abdominal drainage was placed in the Douglas pouch. The duration of the surgery was 190 min, with no transfusion.

**Figure 1:**
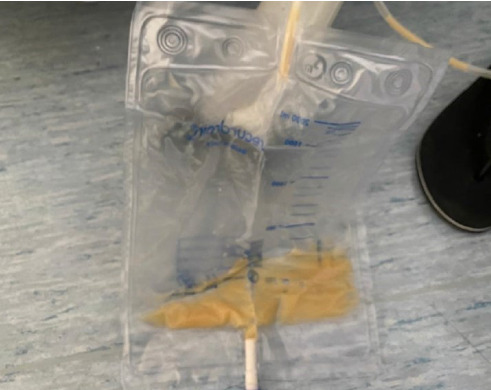
Typical noninfected white milky fluid from surgical drain.

On postoperative Day (POD) 1, the drainage fluid was serous in nature, and the patient begins a water diet. On POD 3, the patient was open to flatus, the drainage was serous in nature, and a liquid diet was started. On POD 5, the drainage fluid turned into not smelly, noninfected white milky fluid (Figure 1). Patients' physical examination and blood tests were normal. Drainage daily amount was 250–300 mL, and the peak drainage amount was 300 mL/day.

An abdominal CT scan was performed, which showed no intra-abdominal collections or anastomotic leak, except for a little free fluid in the Douglas pouch. A triglyceride examination was performed on the drainage fluid, which revealed a value of 400 mg/dl. The culture test of the fluid was negative. Therefore, a diagnosis of chylous leak was made.

The patient was put on a medium chain triglyceride (MCT) diet and parenteral nutritional support.

After 24 h of medical treatment, the drainage fluid reverted to a serous nature and the drainage amount decreased progressively to 50 mL/day. The patient was discharged on POD 16 in good health, after removal of the drain. At 30-day follow-up, the patient was in excellent condition without any symptoms.

## 3. Discussion

CP has a significant impact on the postoperative recovery time of patients. It can lead to malnutrition, electrolyte imbalances, and immunosuppression due to loss of proteins and lymphocytes [1, 2]. In the case presented, the patient developed CP after laparoscopic left hemicolectomy for left-sided colon adenocarcinoma, which emphasizes the importance of recognizing and treating this complication.

The lymphatic system is a one-way drainage system that allows the return of excess interstitial fluids and proteins into the vascular system. The lymph flows from the lymphatic capillaries from the lymphatic vessels and then via the lymph nodes into the lymphatic trunks. The thoracic duct is about 38–45 cm long and begins as dilation called the cisterna chyli anterior to the second lumbar vertebra. The cisterna chyli receives lymph from the right and left lumbar trunks and from the intestinal trunk. CP occurs when these are injured or obstructed [2]. The cisterna chyli is in close contact with the superior mesenteric artery (SMA). During surgical approaches to SMA, e.g., right-colon cancer surgery with central lymphadenectomies or pancreatic resections, injuries can often occur. In left-colon cancer surgery, maybe in the present case, the central lymph node station for the descending and sigmoid colon is located near the inferior mesenteric artery (IMA) on the aorta, the so-called *axilla abdominis of Bacon*.

Many cases present as significant CP with a high triglyceride content in an abdominal drain or an aspirate of postoperative ascites on postoperative resumption of oral intake. The day of onset following surgery ranged from 2 to 8 [1]. In the present case, CP was diagnosed at POD 5.

The standard management approach for CP typically begins with an MCT-based diet, followed, if ineffective, by pharmacologic intervention with somatostatin analogs (e.g., octreotide), then total parenteral nutrition (TPN), and finally surgery if conservative measures fail.

In the intestine the long-chain triglycerides (LCT) are converted into monoglycerides and free fatty acids (FFA) and absorbed as chylomicrons. This explains the high content of triglycerides and the milky and cloudy appearance of the lymph. Short and MCTs, which make up about a third of dietary fat, are absorbed directly via the portal vein system. This fact forms the basis for the use of MCTs as an oral diet in the conservative management of CP [1, 2].

It is important to emphasize that aggressive management of CP is defined in cases of high volume or massive flow, i.e., 150 mL per kg in 24 h (1050 mL in 24 h), which does not correspond to our case. According to these conditions, we think that the appropriate management should be medical, with a standardized approach based on a diet with MCTs.

Surgical intervention is rarely required (about 1% cases) and is usually reserved for patients who do not respond to conservative treatment [1, 6].

As reported [4], CP can be prevented by a routine low-fat diet. Identification of a chylous leak involves CT or MRI lymphangiography.

In addition, the use of real-time ICG lymphangiography should be considered as an option to guide the localization and repair of CP after colonic resection. This involves injecting the fluorescent dye under the serous layer at the proximal end of the anastomosis at the end of surgery [7–12].

The unusually rapid resolution of the CP in this patient could be due to several factors, including the patient's age (younger patients may have a better prognosis, the efficacy of immediate treatment, and possibly, individual variations in lymphatic system response and healing.

## 4. Conclusion

In conclusion CP is a rare but potentially troublesome complication in colorectal surgery, especially in the era of minimally invasive techniques with extensive lymphadenectomies. Recognizing the signs and symptoms and using nonsurgical treatment, including MCT diets and ICG-enhanced fluorescence imaging, can effectively treat this condition. Surgery should only be considered as a last resort when conservative measures fail. Careful preoperative planning and intraoperative techniques can aid in the prevention and early detection of CP and ultimately improve patient outcomes. Fasting and complete parenteral nutrition must be assessed on a case-by-case basis in the treatment of CP after laparoscopic colorectal cancer.

## Data Availability

The data that support the findings of this study are available on request from the corresponding author. The data are not publicly available due to privacy or ethical restrictions.
